# Successful Management of Malignant Glaucoma With Irido-Zonulo-Hyaloidotomy and Complete Pars Plana Vitrectomy

**DOI:** 10.7759/cureus.21679

**Published:** 2022-01-28

**Authors:** Rui Ping Chew, Aliff Irwan Chong, Akmal Haliza Zamli, Julieana Muhammed

**Affiliations:** 1 Department of Ophthalmology and Visual Science, Universiti Sains Malaysia, School of Medical Sciences, Kota Bharu, MYS; 2 Department of Ophthalmology, Hospital Tengku Ampuan Afzan, Kuantan, MYS

**Keywords:** irido-zonulo-hyaloidotomy, pars plana vitrectomy, pseudophakia, aqueous misdirection, malignant glaucoma

## Abstract

Successful irido-zonulo-hyaloidotomy in combination with complete pars plana vitrectomy in malignant glaucoma may lead to better intraocular pressure (IOP) control and a promising visual outcome. We report a case of an 81-year-old woman who presented with a ten-day history of right eye redness and blurring of vision associated with throbbing pain. An ocular examination revealed right-eye visual acuity (VA) of 6/60. The cornea was oedematous with a shallow anterior chamber (AC) and a grade 0 (Shaffer’s grading) by gonioscopy. The intraocular pressure at presentation was 52 mmHg. The optic disc was pink, with a cup-disc ratio of 0.3. Complete pars plana vitrectomy with irido-zonulo-hyaloidotomy was performed in view of poorly controlled intraocular pressure despite aggressive medical therapies, laser treatment, and the Chandler procedure. Postoperatively, the IOP was maintained at mid-teens without intraocular pressure-lowering agents. The visual acuity improved to 6/9. The early decision for irido-zonulo-hyaloidotomy with complete pars plana vitrectomy leads to resolution of malignant glaucoma with a lower relapse risk.

## Introduction

Malignant glaucoma is a form of angle-closure glaucoma, characterised by intraocular pressure (IOP) elevation with a shallow anterior chamber (AC) despite patent peripheral iridotomy (PI), in the absence of posterior segment pathology, causes the forward movement of the lens-iris diaphragm [1]. It is an uncommon condition with a higher incidence rate associated with anterior segment filtration surgery [1,2]. Non-surgical options for treating malignant glaucoma, such as medical therapy with IOP-lowering agents, cycloplegics, and Neodymium: Yttrium aluminium garnet (Nd: YAG) laser posterior capsulotomy and hyaloidotomy, are associated with a high relapse rate of 75-100% [2]. Surgical intervention is often necessitated in refractory cases, and the different approaches that have been previously described include conventional vitrectomy without tunnelling, incomplete vitrectomy either through the anterior or pars plana approach with tunnelling, and pars plana vitrectomy (PPV) with tunnelling [2-5]. We highlight a case with successful treatment of pseudophakic malignant glaucoma with complete PPV with irido-zonulo-hyaloidotomy and restoration of good visual acuity (VA) with no relapse during the 12-month follow-up.

## Case presentation

An 81-year-old woman with underlying hypertension presented with right eye redness and blurring of vision associated with throbbing pain for ten days. She had undergone uneventful cataract surgery in the right eye ten years ago. She had no history of trauma or glaucoma.

Ocular examination revealed the VA of 6/60 in the affected eye improved with pinhole to 6/36 and the left eye VA was 6/18 improved to 6/12 with pinhole. The relative afferent pupillary defect was negative. Examination of the right eye showed injected conjunctiva. The cornea was oedematous with epithelial bedewing. The AC was shallow with gonioscopic findings of Shaffer’s grading grade zero in all four quadrants, and peripheral anterior synaechiae (PAS) was absent. The IOP was 52 mmHg. The left eye examination was unremarkable with a deep anterior chamber, Shaffer’s grading grade four (open) in all four quadrants with an IOP of 12 mmHg. Both eyes' optic discs were pink, with a cup-disc ratio of 0.3. Both the retinae and the maculae were normal.

She was started on an oral carbonic anhydrase inhibitor, topical aqueous suppressants, and topical prostaglandin analogue. A laser PI was performed in the 2 o’clock area. Post-procedure, the PI was patent; however, the AC remained shallow with an IOP ranging from 32 to 40 mmHg. She was then administered with topical cycloplegic, oral glycerol and was subjected to Nd: YAG laser posterior capsulotomy and anterior hyaloidotomy. Unfortunately, the IOP remained high post-procedure.

**Figure 1:**
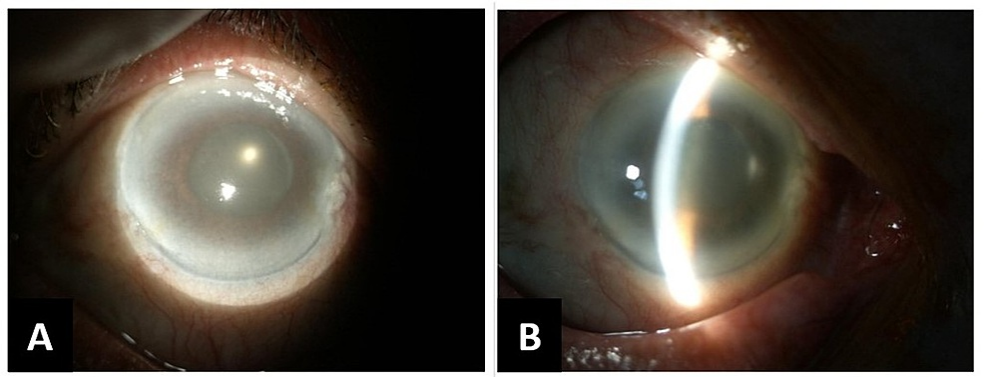
Right eye anterior segment photos post Chandler procedure using slit-lamp biomicroscopy (A) Hazy cornea with a mid-dilated pupil and (B) hazy cornea and shallow anterior chamber can be seen on slit view.

The Chandler procedure was performed subsequently. The IOP was lower than pre-procedure (24 mmHg) but the cornea was still oedematous as shown in Figure 1A. The AC remained shallow from slit-lamp biomicroscopy (Figure 1B).

**Figure 2:**
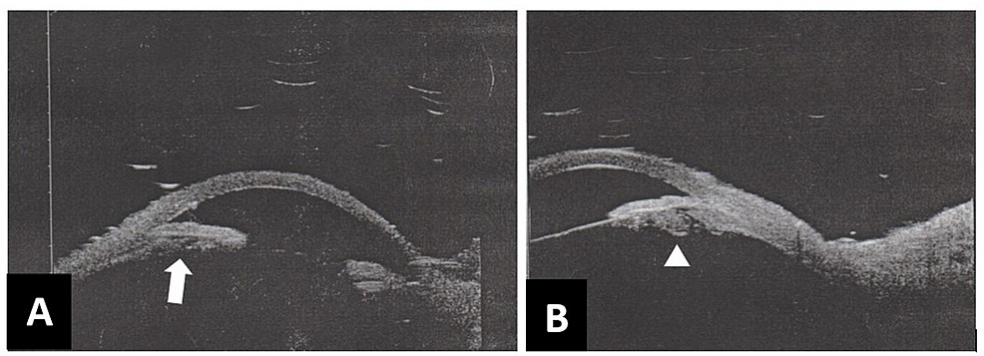
Ultrasound biomicroscopy of the right eye post Chandler procedure (A) Slight bowing of iris, pushing against the trabecular meshwork, and reduced iris-lens distance (white arrow) and (B) hypo-echogenic collection at the ciliary sulcus was seen in (white arrowhead).

Ultrasound biomicroscopy was done after the Chandler procedure, showing a slightly widened angle but persistent bowing of the iris with reduced iris-lens distance and ciliary sulcus collection, as can be seen in Figure 2A-2B.

Two days post Chandler procedure, the IOP rebounded to 30 mmHg, the AC was shallow, and only transiently responded to intravenous mannitol infusion. In view of the refractory response to prior therapies, complete PPV with irido-zonulo-hyaloidotomy was performed on day 14 of diagnosis. A clear corneal incision was made, and a viscoelastic agent was used to reform the AC. Three side ports of 23-gauge were inserted, and a core vitrectomy was done. Iridectomies at the 6 and 10 o’clock positions were created using a vitreous cutter through the anterior approach. Hyaloidotomy followed by zonulectomy and posterior capsulotomy was performed using a vitreous cutter through the pars plana approach under direct visualization through the patent iridectomies. A complete vitrectomy with 360-degree shaving of the vitreous base was performed and deepening of AC was observed at the end of the surgery.

**Figure 3:**
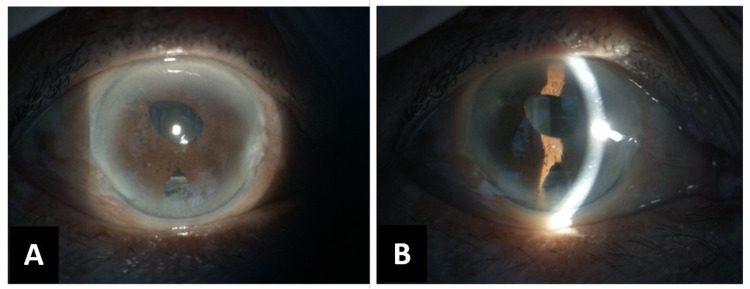
Right eye anterior segment photos using slit-lamp biomicroscopy after complete pars plana vitrectomy with irido-zonulo-hyaloidotomy (A) Clear cornea with patent iridectomies at 6 and 10 o’clock and (B) slit view details the deepened AC.

Post-operatively, the cornea was clear, the AC was deep, and surgical iridectomies at 6 o’clock and 10 o’clock were patent, as shown in Figure 3A-3B. The IOP was maintained in the mid-teens. Her right eye VA improved to 6/9 at her one-month post-operative visit. During sequential follow-ups, the IOP was well controlled and she remained eye-drop free. At her 12-month review, her VA remained 6/9 and the IOP was 10 mmHg.

## Discussion

Malignant glaucoma, also known as aqueous misdirection, is a rare condition. Its incidence ranges from 0.6% to 4% in eyes treated surgically for angle-closure glaucoma, with a higher prevalence after penetrating glaucoma surgeries [6]. Pseudophakia post-cataract surgery has been previously included as one of the possible triggering factors [4,6]. It can be classified into: (i) classic malignant glaucoma, which happens after incisional surgery in patients with primary angle-closure glaucoma; (ii) non-phakic malignant glaucoma, which occurs in patients who have had cataract-removal surgery, which is seen in our case; and (iii) other malignant glaucoma syndromes, which have sporadic occurrence and may be associated with laser, miotic agents, and trabeculectomy bleb needling [1].

One of the theories behind the pathogenesis of malignant glaucoma is that the laxity of lens zonules coupled with vitreous pressure leads to forward lens movement [7]. The higher the pressure in the posterior segment, the more firmly the lens is held forward, and the vicious cycle repeats. A passage through the posterior capsule must be created and the anterior vitreous be removed to disrupt the anatomic relationship between the ciliary processes, the lens, and the anterior vitreous face [1]. 

Our patient presented with a VA of 6/60, a closed-angle, and an IOP of 52 mmHg. A laser PI was performed to exclude a possible pupillary block angle closure mechanism. Pseudophakic malignant glaucoma was diagnosed when the AC remained shallow despite a patent iridotomy. Topical cycloplegic, oral, and topical IOP-lowering agents, as well as an intravenous hyperosmotic agent, were initiated, but the IOP remained high. Subsequent Nd: YAG laser posterior capsulotomy and anterior hyaloidotomy procedures also failed to reduce the IOP, although a transient deepening of the central AC depth was observed. In malignant glaucoma, Nd: YAG laser posterior capsulotomy in conjunction with hyaloidotomy aims to disrupt the posterior capsule and anterior hyaloid membrane to allow communication between the drainage angle of the AC and vitreous cavity. However, the relapse rate is as high as 75% [3]. Another option is the Chandler procedure, which was first described in 1964 and aims to remove the anterior hyaloid face, thus redirecting the posterior segment positive pressure [7]. Our patient had the Chandler procedure completed on day 5 of treatment after failing medical and laser therapies. However, the IOP was still uncontrolled post-procedure.

The vast variability of surgical options for malignant glaucoma has been reported with variable success and a relapse rate. Options include lens extraction for the phakic patient, conventional vitrectomy without tunnelling, anterior vitrectomy with tunnelling, and vitrectomy with tunnelling. Conventional vitrectomy without tunnel construction theoretically does not reverse the pathology as the absence of communication between the AC and posterior vitreous cavity will lead to future build-up of posterior vitreous pressure and a relapse rate of 75% was described, although initially success might be observed [2].

Irido-zonulo-hyaloido-vitrectomy can be performed via the anterior or pars plana approach [8]. For the anterior approach, a corneal incision parallel to the iris plane is created. Iridectomy is then performed, followed by passing the vitreous cutter through the iridectomy for subsequent hyaloidectomy and anterior vitrectomy. The Pars plana approach to vitrectomy otherwise permits a better field of view, wider access to allow disruption of anterior hyaloid, and has a lower recurrence rate as compared to the anterior approach. This is postulated to be due to satisfactory vitreous base removal through the pars plana approach coupled with the feasibility of total vitrectomy to prevent secondary blockage by the residual vitreous [3]. Irido-zonulectomy in combination with incomplete PPV, which includes anterior vitrectomy, core vitrectomy, or partial vitrectomy, has observed a higher percentage of relapse during follow-up [3]. Anterior vitrectomy performed via anterior approach with tunnel creation serves the benefit of allowing the anterior segment surgeon to manage refractory malignant glaucoma, especially in the setting short of vitreoretinal expertise. However, a relapse rate ranging from 40% to 66% was observed after a mean follow-up period of 50 days to four years, likely owing to the remnant of vitreous interfering with the patency of the antero-posterior segment tunnel [2,8].

On day 14, after the diagnosis of malignant glaucoma, a 23-gauge complete PPV with irido-zonulo-hyaloidotomy was performed on our patient. Similar procedures were described and the majority of patients observed no or very low recurrence throughout the follow-ups [2,4,5]. During the post-operative period, the IOP was maintained at mid-teens without IOP-lowering eye drops. Gonioscopy revealed an open angle and the absence of PAS. Our patient was asymptomatic, had no recurrence of AC shallowing, and remained eye-drop free throughout the 12-month follow-up.

The prognosis of complete PPV with irido-zonulo-hyaloidotomy is promising and favours a low relapse rate. In this 10-year retrospective study, a 90.3% anatomical success rate with deepening of AC after PPV and irido-zonulo-hyaloidectomy was described [5]. Case reports by Debrouwere et al. reported none of the eyes with relapse after this procedure during two-month follow-ups [2]. Raj et al. found only two out of 16 eyes that had undergone similar surgery required another IOP lowering surgery [4].

The stepladder approach for the treatment of malignant glaucoma is commonly practiced, starting with medical therapies, followed by laser therapies, and finally, vitrectomy. However, a longer interval of more than a month from diagnosis to the timing of vitrectomy has been shown to significantly reduce the post-operative success rate and visual outcome [9]. In our case, the early decision to complete vitrectomy and irido-zonulo-hyaloidotomy in two weeks carries a good visual outcome as well as prevents relapse secondary to PAS development in chronic refractory malignant glaucoma.

## Conclusions

Malignant glaucoma is a relatively rare condition. Thus, the variability of treatment modalities and approaches makes it difficult to decide on the exact timing for complete PPV with irido-zonulo-hyaloidotomy. We believe that this option will add value to the small number of reported literature on the successful management of malignant glaucoma with early complete vitrectomy alongside irido-zonulo-hyaloidotomy during an early course of treatment with a promising visual outcome.
